# Effect of intraoperative cold solution irrigation to reduce postoperative pain in knee osteoarthritis patients who underwent unilateral primary total knee arthroplasty: a double-blinded randomized controlled trial

**DOI:** 10.1186/s12891-024-07732-3

**Published:** 2024-08-01

**Authors:** Suwis Charoenwisetsin, Vorakran Jiranantarat, Paphon Hirunyachoke, Pacharapol Udomkiat

**Affiliations:** 1 Department of Orthopaedic Surgery, Faculty of Medicine Siriraj Hospital, Mahidol University, Bangkok, Thailand; 2 Department of Anesthesiology, Faculty of Medicine Siriraj Hospital, Mahidol University, Bangkok, Thailand

## Abstract

**Purpose:**

To compare the postoperative pain score, opioid consumption, and blood loss in knee osteoarthritis patients who underwent unilateral primary total knee arthroplasty with and without intraoperative cold solution irrigation.

**Method:**

In total, 70 knee osteoarthritis patients were randomly included in the study and allocated into 2 groups. The first group was irrigated intraoperatively with a cold solution and the second group was irrigated intraoperatively with a room-temperature solution.

**Results:**

The cold solution group showed significantly lower pain scores (numerical rating scale, NRS) at 28 h postoperatively (*p* = 0.047). There were no significant differences in opioid consumption or blood loss between the groups.

**Conclusions:**

Intraoperative cold solution irrigation in unilateral primary total knee arthroplasty patients may provide the benefit of early postoperative pain reduction for up to 28 h but has no effect in terms of reducing opioid consumption or blood loss.

**Trial registration:**

The trial was registered in the Thai Clinical Trials Registry (TCTR) Trial registration number ID: TCTR20200706001 on 06/07/2020.

**Supplementary Information:**

The online version contains supplementary material available at 10.1186/s12891-024-07732-3.

## Background

Total knee arthroplasty (TKA) is the gold standard treatment for knee osteoarthritis patients who have failed conservative treatment to decrease pain and disability [1, 2]. Postoperative pain after TKA is usually at a maximum of 24 to 48 h postoperatively [3]. Inadequate pain control can cause delayed recovery and complications, such as deep vein thrombosis, wound problems, and longer hospital stays [4].

Currently, the best method for postoperative TKA pain control is unknown [5–7], but the present general concept for postoperative pain control is multimodal analgesia treatment, which uses multiple medications and physical modalities to maximize the analgesic effects and minimize adverse effects [8].

Irrigation is a standard step during TKA operations [9, 10]. Irrigation has various benefits, including washing off residual bone and soft tissue debris to reduce bacterial accumulation and reduce third-body wear [11]. Accordingly, the US Centers for Disease Control and Prevention (CDC) in 2017 and the World Health Organization (WHO) in 2018 recommended diluted povidone–iodine solution irrigation intraoperatively to reduce surgical site infection [12, 13], but there is no consensus on the optimal volume of irrigation in TKA [14].

Cryotherapy is another common modality used in acute postoperative pain control. The physiology of cryotherapy consists of vasoconstriction and hemostasis for decreasing inflammation and swelling which in turn helps delay pain signal transmission and muscle spasm [15, 16]. Previous studies have reported various methods for cryotherapy applications, such as ice, a cold pack, and cold flow devices. The therapeutic effects of cryotherapy include decreased pain score, analgesic consumption, and blood loss, and improved functional knee score [17–21]. A meta-analysis of postoperative cryotherapy studies also found small benefits in reducing VAS at 48 h postoperatively, and blood loss (225 mL), and an improvement in ROM at discharge [22–24]. The main obstacle to effective postoperative cryotherapy is the barrier between deep-seated injured tissue and the superficial cold application, such as the soft tissue thickness, surgical wound bandage, and the inability to disperse cold application due to the sparing of some areas that are prone to complications, such as the patella area and popliteal fossa. One study found superficial cold could penetrate deep tissue to only 4 cm in depth [25]. Consequently, there is interest in further studying intraoperative cryotherapy to be applied cold directly into deep injured tissue. Also, one study found that intraoperative cryotherapy in knee arthroscopy did not offer benefits, and one explanation for this could have been the minor degree of soft tissue injury from the arthroscopic procedure utilized in the study [26].

Regarding the use of cryotherapy in TKA patients, there was a single study by Li et al. in 2016 that used cold saline mixed with 0.5% epinephrine irrigation intraoperatively and compared this with room-temperature saline irrigation. The study authors found that cold saline could reduce VAS at 4–24 h postoperatively, and reduce analgesic consumption and blood loss. However, the results from that study may have been affected by the addition of epinephrine and the unusually prolonged irrigation time [27].

Our study aimed to evaluate whether a combination of two previously mentioned routine steps in perioperative TKA care, which is an intraoperative irrigation with a cold solution, could improve the early postoperative TKA results. The research compared intraoperative cold solution irrigation with room temperature solution irrigation without additional drugs in terms of the postoperative pain score, opioid consumption, and blood loss in both procedures.

## Materials and methods

### Study design

**Fig. 1:**
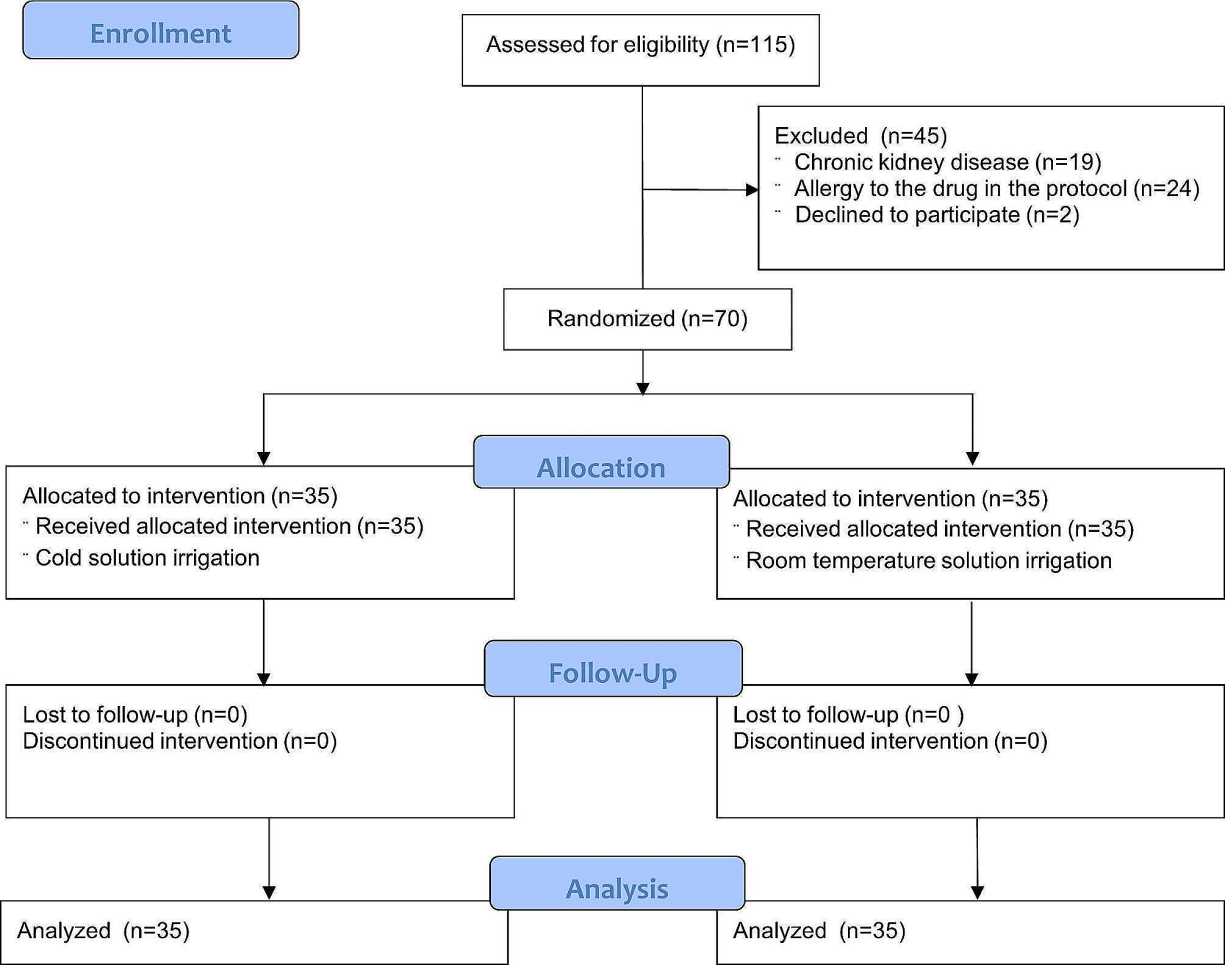
Consolidated standards of reporting trials (CONSORT) flow diagram for patient enrollment in the study

This was a double-blinded randomized controlled study that was performed at Siriraj Hospital, Mahidol University from July 2020 to Jan 2023, approved by Siriraj Institutional Review Board Certificate of Approval *COA no. Si 544/2020* and was registered in the Thai Clinical Trials Registry (TCTR) Trial registration number: *TCTR20200706001 on 06/07/2020*. The inclusion criteria were knee osteoarthritis patients who underwent unilateral primary TKA and who were 45 years old or older and gave their informed consent to participate. The exclusion criteria were patients who had cold allergy/cold intolerance, allergy to any medication used in the protocol (tranexamic acid, ketorolac, levobupivacaine, morphine, parecoxib, eperisone, acetaminophen, codeine, pregabalin, marcaine, heavy marcaine, dexamethasone), had an eGFR lower than 50 mL/min/1.73 m^2^, received anesthesia other than a spinal block with an adductor canal block, psychiatric disorder, cognitive impairment, bleeding disorder, unable to withhold antiplatelet/anticoagulant before surgery except low dose aspirin, needed an additional procedure other than the standard cruciate-retaining total knee arthroplasty, and/or refused to give informed consent or rejected participation. The patients were randomized into 2 groups by computer-generated mixed block-sized randomization (block sizes = 4, 6, 8). The sequence of randomization was concealed with an opaque sealed envelope that was opened intraoperatively. The patients and the assessors were blinded. The CONSORT flow diagram for patient enrollment in this study was shown in Fig. 1. The sample size was calculated based on the study by Li et al., using an alpha level of 0.05 and a beta level of 0.05. The calculation was performed using a pre-procedure NRS score of 3.0 with a standard deviation (SD) of 2, and a post-procedure NRS score of 2.2 with an SD of 2.

### Surgical technique and intervention

In total, 70 patients were included in the study. Before surgery, the patient’s demographic data were collected, including age, sex, BMI, baseline pain score (numerical rating scale, NRS 0–10), baseline opioid consumption, and hemoglobin and hematocrit levels. The Numerical Rating Scale (NRS) is an 11-point scale where the endpoints represent the extremes of no pain and the worst pain imaginable [28]. In our study, the NRS was delivered verbally to the participants. All the patients received antibiotics prophylaxis and tranexamic acid 750 mg intravenous 30 min before undergoing the operation. A tourniquet was applied and the pressure was set at 300 mmHg. Unilateral primary TKA was performed by a single adult reconstructive orthopedic surgeon under spinal plus an adductor canal block in every case. The patients were kept warm during the operation with a heater. After skin preparation and draping in a sterile fashion, a midline incision was done with a medial parapatellar approach. A P.F.C. sigma cruciate-retaining prosthesis (Johnson DePuy Synthes, USA) was used for all the patients. After bone cut and gap balancing were done, all the patients received periarticular injections with a similar regimen cocktail (ketorolac 30 mg + levobupivacaine 100 mg diluted with NSS 20 mL) and the same amount at each location (infrapatellar fat pad, medial and lateral gutter periosteum, medial and lateral collateral ligament, posterior capsule, and quadriceps muscle); then the opaque sealed envelope that contained the randomized sequence was opened. The patients were divided into 2 groups: the cold solution irrigation group and the room temperature solution irrigation group. The cold solution irrigation group was irrigated with a mixture of NSS 1000 mL + 10% povidone–iodine 250 mL, with both stored at 2–8 °C. The room temperature solution irrigation group was irrigated with a mixture of NSS 1000 mL + 10% povidone–iodine 250 mL, with both stored at room temperature.

The irrigating solutions were stored in a nearby temperature-controlled refrigerator/shelf and transported to the operating room just before the irrigation step. After mixing the solution in the operative field, 50 mL of the mixed solution was brought out to measure its actual temperature using a digital thermometer (Extech 39240, Extech Instruments, USA). The irrigation was divided into two periods: before and after the cemented components implantation. The mixed solution was soaked intraarticularly during wound closure and was suctioned before the closure was done. A vacuum drain was placed through a superolateral aspect of the patella. The total irrigation time was recorded. The perioperative body temperature of all the patients was recorded in both the waiting room and recovery room. The vacuum drain was clamped for 3 h, then released and removed at 48 h postoperatively. All the patients received postoperative analgesics around the clock with the same regimen, except for intravenous opioids for breakthrough pain. NRS 0–10 was recorded every 4 h until 72 h postoperatively. Mechanical DVT prophylaxis was applied to all the patients. Two cold packs that were stored at 0 °C for at least 2 h were applied for 20 min every 2 h until 72 h postoperatively. Rehabilitation was initiated on postoperative day 1 with the same program for all the patients. Hemoglobin and hematocrit levels were routinely taken on postoperative day 1 and additionally on day 3 for calculating the estimated blood loss (Mercuriali formula) and hemoglobin difference compared to baseline. All the patients were discharged after achieving knee flexion of at least 90 degrees, NRS ≤ 3, and once they were able to perform basic ADL with a gait aid. All complications during the admission period were recorded.

### Estimated blood loss calculation

The Nadler equation was used to calculate blood volume for inputting in the Mercuriali formula for males and females:

Male; Blood volume = (0.3669 × H3) + (0.03219 × W) + 0.6041

Female; Blood volume = (0.3561 × H3) + (0.03308 × W) + 0.1833

where H is the patient’s height in meters, and W is the patient’s weight in kilograms.

The estimated RBC loss was calculated via the Mercuriali formula using the blood volume figures calculated above:

Estimated RBC loss = [Blood volume × (Preop Hct - Postop Hct day3)] +

the volume of transfused RBC (Blood volume in milliliters, Hct in decimal, the volume of transfused RBC in milliliters of RBC).

The estimated RBC loss was then converted into the estimated blood loss in milliliters by dividing by the mean Hct (mean Hct = (Preop Hct + Postop Hct day3) ÷ 2).

### Statistical analysis

We analyzed descriptive statistics with the mean ± SD for continuous variables and percentage for categorical variables. The independent t-test/Mann–Whitney U-test and chi-square test/Fischer’s exact test were used to compare variables between the 2 groups. Inferential statistics, for the primary outcome (NRS) between the 2 groups, were analyzed with an independent t-test at each time point. The secondary outcomes (total opioid consumption, estimated blood loss, hemoglobin difference, and total drain content) were analyzed with an independent t-test. The statistical analyses were performed using SPSS Statistics for Windows, version 18.0 software (SPSS, Inc., Chicago, IL, USA). Statistical significance was defined as a *p*-value less than 0.05.

## Results

70 patients in the study were randomized into 2 groups: 35 participants in the cold solution group and 35 participants in the room temperature solution group. There was no dropout of participants in this study. The average solution temperature in the cold group was 11.1 °C while it was 18.6 °C in the room temperature group. The total irrigation time was estimated at 2–3 min.

There were no significant differences in the baseline characteristics between the 2 groups, including age, BMI, gender, baseline NRS, and baseline opioid consumption (Table 1).

**Table 1:** Baseline characteristics of the enrolled patients

Demographic data	Cold irrigation (*n* = 35)	Room temperature (*n* = 35)	*p*-value
Age	68.14 ± 5.54	70.46 ± 7.25	0.138
BMI (kg/m^2^)	26.77 ± 3.67	26.76 ± 4.10	0.992
Gender			
Male	5 (62.5%)	3 (37.5%)	0.355
Female	30 (48.4%)	32 (51.6%)	
Side			
Right	17 (48.6%)	18 (51.4%)	1.000
Left	18 (51.4%)	17 (48.6%)	
OA			
Primary	31 (47%)	35 (53%)	0.114
Secondary	4 (100%)	0 (0%)	
Baseline pain score at rest	1.21 ± 2.10	0.84 ± 2.07	0.481
Baseline pain score while walking	5.31 ± 1.84	4.79 ± 2.5	0.356
Operative time (min)	85.63 ± 18.79	76.09 ± 0.33	0.026*
Intra-op blood loss (ml)	13.86 ± 16.81	14.29 ± 11.32	0.901^b^
Pre-op body temp (°C)	36.37 ± 0.33	36.40 ± 0.31	0.682^b^
Post-op body temp (°C)	35.89 ± 0.31	35.91 ± 0.28	0.749
Baseline opioid (MO equivalent (mg))	0.185 ± 0.82	0.523 ± 1.91	0.368^b^

*sig. *p*-value < 0.005, ^b^ Mann–Whitney U test analysis was performed because the data were non-normally distributed

**Fig. 2:**
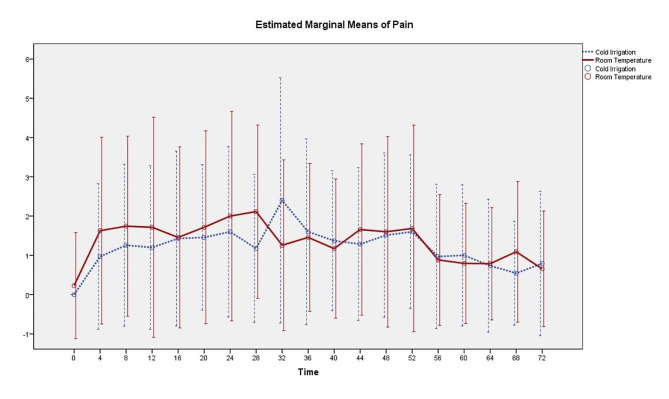
Estimated marginal mean NRS for the two groups of patients

The NRS of the cold solution group was slightly lower in the early postoperative period up to 28 h (Fig. 2), but significantly lower only at 28 h after the operation (*p* = 0.047). There were no significant differences in postoperative opioid consumption between the 2 groups on postoperative days 1, 2, and 3 (*p* = 0.869, *p* = 0.394, and *p* = 0.329 respectively) (Table 2). There was no significant reduction in the calculated blood loss in the cold solution group (*p* = 0.761). Also, there were no significant differences in the hemoglobin difference (*p* = 0.635), drain content (*p* = 0.391), and amount of packed red cell transfusion (*p* = 0.463) (Table 3). The cold solution groups had a significantly longer operative time (*p* = 0.026). The patients in the cold solution group had a significantly shorter length of hospital stay (*p* = 0.039). No patients in either group developed wound complications or surgical site infection. Also, no hypothermia developed in the cold solution group.

**Table 2:** Postoperative pain scores of the two groups

	Group		Mean difference (95% CI)	*P*-value
	Cold solution	Room temp solution		
Pain (NRS)				
0 h	0 ± 0	0.25 ± 1.41	-0.25 (-0.79, 0.29)	**0.317** ^**b**^
4 h	1.07 ± 1.99	1.69 ± 2.47	-0.62 (-1.79, 0.56)	**0.153** ^**b**^
8 h	1.00 ± 1.66	1.88 ± 2.35	-0.88 (-1.91, 0.17)	**0.385** ^**b**^
12 h	1.39 ± 2.23	1.75 ± 2.91	-0.36 (-1.71, 0.99)	**0.603** ^**b**^
16 h	1.61± 2.39	1.47 ± 2.38	0.14 (-1.10, 1.38)	**0.858** ^**b**^
20 h	1.57 ± 1.95	1.72 ± 2.56	-0.15 (-1.34, 1.04)	**0.814** ^**b**^
24 h	1.71 ± 2.23	2.13 ± 2.76	-0.41 (-1.72, 0.90)	**0.724** ^**b**^
28 h	1.25 ± 1.99	2.25 ± 2.26	-1.00 (-2.11, 0.11)	**0.047** ^*b^
32 h	2.68 ± 3.38	1.38 ± 2.24	1.30 (-0.21, 2.82)	**0.134** ^**b**^
36 h	1.82 ± 2.54	1.56 ± 1.93	0.26 (-0.90, 1.42)	**0.852** ^**b**^
40 h	1.25 ± 1.92	1.22 ± 1.83	0.03 (-0.94, 1.00)	**0.539** ^**b**^
44 h	1.18 ± 2.07	1.59 ± 2.21	-0.42 (-1.53, 0.70)	**0.504** ^**b**^
48 h	1.61 ± 2.27	1.75 ± 2.49	-0.14 (-1.38, 1.09)	**0.942** ^**b**^
52 h	1.79 ± 2.08	1.81 ± 2.72	-0.03 (-1.29, 1.24)	**0.674** ^**b**^
56 h	0.71 ± 1.58	0.91 ± 1.71	-0.19 (-1.05, 0.66)	**0.894** ^**b**^
60 h	1.07 ± 1.92	0.81 ± 1.58	0.26 (-0.65, 1.16)	**0.857** ^**b**^
64 h	0.82 ± 1.83	0.78 ± 1.45	0.04 (-0.81, 0.89)	**0.365** ^**b**^
68 h	0.57 ± 1.40	1.13 ± 1.81	-0.56 (-1.40, 0.29)	**0.151** ^**b**^
72 h	0.82 ± 1.87	0.66 ± 1.47	0.17 (-0.70, 1.25)	**0.992** ^**b**^

*sig. *p*-value < 0.005, ^b^ Mann–Whitney U test analysis was performed because the data were non-normally distributed

**Table 3:** Comparison of the differences in opioid consumption, calculated blood loss, drain content, packed red cell transfusion, and length of hospital stay between the two groups

	Group		Mean difference (95% CI)	*P*-value
	Cold Irrigation	Room temperature		
Morphine (mg)				
Day 1	2.01 ± 3.34	1.64 ± 2.43	0.37 (-1.02, 1.76)	**0.869** ^**b**^
Day 2	1.90 ± 2.39	1.80 ± 3.76	0.10 (-1.40, 1.60)	**0.394** ^**b**^
Day 3	1.00 ± 1.50	0.77 ± 1.31	0.23 (-0.44, 0.90)	**0.329** ^**b**^
Total morphine	4.33 ± 6.16	4.33 ± 6.82	0.79 (-2.31, 3.89)	**0.343** ^**b**^
Hb (g/dL)				
Pre-op	12.53 ± 1.13	12.60 ± 1.50	-0.17 (-0.82, 0.49)	0.62
Post-op Day 1	10.85 ± 1.16	10.60 ± 1.44	0.15 (-0.50, 0.80)	0.652
Post-op Day 3	10.36 ± 0.95	10.42 ± 1.18	-0.03 (-0.57, 0.50)	0.897
Hct (%)				
Pre-op	38.46 ± 3.40	38.96 ± 4.21	-0.73 (-1.70, 2.19)	0.455
Post-op Day 1	32.94 ± 3.22	32.53 ± 4.20	0.25 (-1.70, 2.19)	0.8
Post-op Day 3	31.56 ± 2.80	31.95 ± 3.62	-0.46 (-2.12, 1.19)	0.576
Total drain (ml)	329.53 ± 173.86	320.16 ± 260.12	9.37 (-101.76,)	0.391^b^
PRC transfusion (ml)	26.50 ± 84.03	51.42 ± 124.94	-24.92 (-78.41, 28.57)	0.463^b^
Hb difference (g/dL)	2.04 ± 0.82	2.17 ± 1.15	-0.13 (-0.64, 0.377)	0.635^b^
Calculated blood loss (ml)	687.18 ± 273.26	712.37 ± 372.27	-25.19 (-190.50, 140.12)	0.761
Length of Stay (days)	5.47 ± 0.62	5.89 ± 0.90	-0.415 (-0.79, -0.04)	**0.039** ^*b^

*sig. *p*-value < 0.005, ^b^ Mann–Whitney U test analysis was performed because the data were non-normally distributed

## Discussion

The peak postoperative pain scores occurred at 24 to 32 h in our study, which concorded with the literature [22, 23, 27]. Our study also demonstrated a lower early postoperative NRS up to 28 h, with the mean difference in NRS at 28 h reaching the MCID, as defined by P. S. Myles et al. [29]. These results were comparable with the results of a previous study [27] that reported a significantly lowered postoperative VAS from 4 to 24 h, albeit our result was significant only at 28 h postoperatively. The possible explanations for this are the following:

### The lower average pain score of our study

Our study’s intensive postoperative pain control protocol, which included a combination of multiple analgesics and physical modalities, may have suppressed baseline postoperative pain and masked the modest effect of intraoperative cryotherapy. The NRS at 28 h was significantly lower in the cold solution group because that time point was the highest pain period, which may indicate the effect of intraoperative cryotherapy.

### The irrigation time and volume

In our study, the average irrigation time in both groups was about 2 to 3 min, which replicated the real-life practice in most orthopedics institutes. The shorter period of intraoperative cryotherapy and much lower irrigation volume in our study than in the study of Li et al. [27] (1200 ml vs. 4000 ml) may have decreased the analgesic effect.

### Temperature loss of the irrigation solution

Although we stored the irrigation solution in the closest temperature-controlled refrigerator and transferred it to the operative field as late as possible during the irrigation period, some inevitable temperature change of the irrigation solution toward room temperature must have occurred and may have blunted the effect of the intraoperative cryotherapy.

Opioid consumption was not significantly different in both groups. Our study’s lower average pain score, with an NRS lower than 3 for most of the early postoperative period, may have been the reason for the only small amount of rescue analgesics consumed in both groups.

The calculated blood loss, hemoglobin difference, and drain content showed no significant differences between both groups, which contrasts with the findings from Li et al.’s study [27] that showed a reduction in postoperative hemoglobin and drainage output in the cold solution group. A major difference in the irrigation solution of our study was that no epinephrine was added to an irrigation solution in both groups, which contrasts with Li et al.’s study, where epinephrine was used in the cold solution group. Moreover, our study did not administer epinephrine through a drainage tube after the operation. The lack of a hemostatic effect of the epinephrine in the intraoperative and immediate postoperative periods may have been the reason for the similar blood loss parameters in both groups. We concluded from the results that intraoperative cold solution irrigation did not affect postoperative blood loss. No wound complications or surgical site infections were observed in any patient in either group in our study. Finally, the cold solution group patients had a statistically significantly shorter length of hospital stay. Although it was only by one day, this could still reduce the total cost and improve patient satisfaction greatly.

There are limitations of our study to note, including the small sample size, which may have meant the study lacked the power to detect minor differences. Also, our study did not have a method to maintain the temperature of the irrigation solution during the irrigation period, so some loss of cold during the irrigation period was inevitable. A small amount of irrigation solution and a brief irrigation period may minimize the effect of this on intraoperative cryotherapy.

Moreover, the results also showed a statistically significant difference in postoperative pain scores between the two groups at some time points. Therefore, it confirmed the analgesic effect of intraoperative cold solution irrigation. Further study with higher power may reveal the obvious analgesic effect of intraoperative cryotherapy, and the functional outcome also needs to be further investigated.

A notable limitation of our study is the use of a tourniquet during surgery. According to recent literature by Ahmed et al. [30], the routine use of tourniquets in TKA should be reconsidered due to benefits like reduced postoperative pain. The inclusion of a tourniquet in our study may have influenced our results. Future studies without the use of a tourniquet should be conducted to accurately assess the true effects of cold solution irrigation on postoperative outcomes.

## Conclusions

Intraoperative cold solution irrigation in unilateral primary total knee arthroplasty patients may benefit from early postoperative pain reduction for up to 28 h but has no effect on the reduction of opioid consumption or blood loss. Intraoperative cold solution irrigation is a safe method to improve postoperative pain after total knee arthroplasty.

### Electronic supplementary material

Below is the link to the electronic supplementary material.

Supplementary Material 1

## Data Availability

The data that support the findings of this study are not publicly accessible due to sensitivity concerns and ethical considerations, as mandated by the ethics approval and consent procedures outlined in the Siriraj Institutional Review Board’s Certificate of Approval, COA no. Si 544/2020. Nevertheless, these datasets are available from the corresponding author on reasonable request.

## Declarations

### CONSORT

Our study adheres to CONSORT guidelines

## Abbreviations

TKA: Total Knee Arthroplasty
NRS: Numerical Rating Scale
VAS: Visual Analog Scale
